# Comparison between Piezoelectric and Piezoresistive Wearable Gait Monitoring Techniques

**DOI:** 10.3390/ma15144837

**Published:** 2022-07-12

**Authors:** Zhiyuan Zhang, Zhenyu Xu, Wenbin Chen, Shuo Gao

**Affiliations:** School of Instrumentation and Optoelectronic Engineering, Beihang University, Beijing 100191, China; exzzy32@163.com (Z.Z.); 20373962@buaa.edu.cn (Z.X.); 19376299@buaa.edu.cn (W.C.)

**Keywords:** piezoresistive material, gait analysis, plantar stress detection

## Abstract

Insole plantar stress detection (PSD) techniques play an important role in gait monitoring. Among the various insole PSD methods, piezoelectric- and piezoresistive-based architectures are broadly used in medical scenes. Each year, a growing number of new research outcomes are reported. Hence, a deep understanding of these two kinds of insole PSD sensors and state-of-the-art work would strongly benefit the researchers in this highly interdisciplinary field. In this context, this review article is composed of the following aspects. First, the mechanisms of the two techniques and corresponding comparisons are explained and discussed. Second, advanced materials which could enhance the performance of current piezoelectric and piezoresistive insole prototypes are introduced. Third, suggestions for designing insole PSD prototypes/products for different diseases are offered. Last, the current challenge and potential future trends are provided.

## 1. Introduction

Among all the wearable medical devices, gait-analysis-based insole systems are more suitable for chronic disease diagnosis and rehabilitation. Neurologic and orthopedic chronic diseases are caused by lesions of the central nervous system (CNS), peripheral nerves (PNs), and orthopedic limbs. Gait features are comprehensive results generated from the coordination of CNS, PN, and lower limbs [1,2,3]. Thus, gait features can contribute to the analysis and diagnosis of specific diseases.

Among all the features of gait, plantar stress distribution is most commonly used for diseases analysis, for two main reasons: First, plantar stress distribution is closely associated with multiple chronic diseases. For instance, patients with diabetic feet place more pressure in the big toe and heel, whereas patients with Parkinson’s disease (PD) have lower peak plantar pressure [4,5]. Additionally, the high-amplitude response in PSD can facilitate diagnosis easier. For example, one clinical manifestation of PD is that the peak plantar pressure decreases by approximately 40% [6]. Second, the calculation of other spatial and temporal gait features is based on the plantar pressure. For example, the gait frequency and velocity are calculated by measuring the time interval between the adjacent appearance of peak pressures [7,8].

Generally, the mainstream techniques used for PSD can be classified into five categories: piezoresistive, piezoelectric, capacitive, resistive, and inductive methods. Among them, piezoresistive- and piezoelectric-related sensors are the most frequently used in insole systems [9,10] for the following reasons: Piezoresistive PSD sensors normally have simple structures, high sensitivity, and high-amplitude responses. Piezoelectric PSD sensors are passive and provide multiple-dimensional detection.

**Figure 1:**
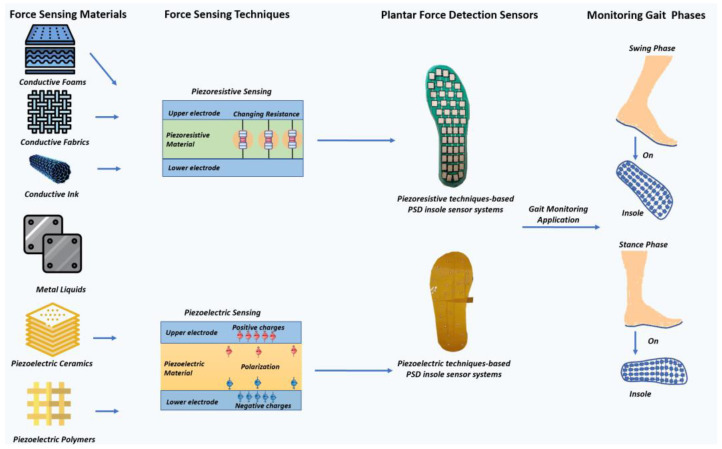
Mechanisms, representative types, and correspondent insole systems or PSD sensors composed of piezoelectric and piezoresistive materials. Piezoresistive system is produced in [11] and piezoelectric system is produced in [12].

During the sensor development procedure (as shown in Figure 1), selecting suitable materials and designing a reasonable sensor layout are very important, especially when the product is for biomedical applications. Therefore, in-depth explanation of essentials of the piezoresistive and piezoelectric PSD sensors from aspects of intrinsic principles, material properties, the relationship between the sensor layout and different diseases, and the state-of-the-art prototypes and products are strongly required, but are often omitted in previous reviews, as this field is highly interdisciplinary. In this context, this article is created. Compared to the current works, this article specifically contributes the following points:

- introducing the most advanced force sensitive materials and discussing their potential integration into the current insole PSD sensors;
- conducting a detailed comparison of piezoresistive and piezoelectric based insole PSD architectures;
- explaining diseases features and providing reasonable advice for insole PSD prototypes/products design for diverse neural-skeleton diseases.

The above fruitful results indicate that piezoresistive and piezoelectric sensing materials have been applied in PSD and force sensing. However, these sensors still present predicaments from the materials aspect and PSD aspects.

To conduct this work, we searched references from IEEE, Wiley, MDPI, etc. Keywords included “chronic diseases”, “insole systems”, “piezoresistive sensors”, “piezoelectric sensors”, etc. The gathered references were classified into three aspects according to their research focus. Medical essays provide us with gait features like higher peak plantar pressure. Research papers on current piezoresistive and piezoelectric PSD sensors present us with the mechanisms, applications, and features of the two techniques. Research papers on new advanced materials published in the last five years provide us with choices of potential force-sensing materials in PSD sensors.

As for the methodology of this essay, in Section 2, we classify the materials used for each sensing technique, and we describe the working principles of each type of material according to the literature. In Section 3, we review literature from three aspects: current PSD products, advanced materials for force sensing, and potential applications of PSD-based disease diagnosis. By reviewing the above information, we compare piezoelectric and piezoresistive wearable gait monitoring techniques in terms of suitable materials, sensor performance, and medical applications. In Section 4, we analyze the current limitations of the above studies, which we consider from application and sensing materials perspectives. Therefore, based on these challenges and the development trends in medical wearable devices, we think that multi-sensing-based self-calibration and digital twin models can be combined with these two techniques.

## 2. Mechanisms of Piezoresistive and Piezoelectric Techniques

These techniques are based on the characteristics of materials. Various materials can enhance the performance of sensors in different aspects.

For piezoresistive sensors, conductive polymers such as thermoplastic polyurethane (TPU) [13], fiber-reinforced polymer (RFP) [14], and Parylen-C polymer film [15] can be used to increase sensor flexibility. Mixtures of carbon nanotubes and metal particles [11,16,17,18] can improve the sensing range and sensitivity of the sensors. Metal liquids such as E-GaIn-based piezoresistive PSD sensors can detect shear stress [19].

For piezoelectric sensors, using piezoelectric ceramics such as ethyl cellulose-poly lead zirconate titanate piezoelectric ceramics (ECS-PolyPZT) [20] and PZT-ferroperm [21] as the sensing material can produce high piezoelectric coefficients. Using piezoelectric polymers such as polyvinylidene fluoride (PVDF) [12,22] and polyacrylonitrile/barium titanate (PAN-C/BTO) [23] can enhance sensor flexibility and enable three-dimensional detection.

Though these materials possess distinct virtues, they still share common sensing mechanisms. Thus, to compare these two techniques, we first reviewed the mechanisms of techniques according to the classification of the materials.

### 2.1. Piezoresistive Sensing Mechanisms and Suitable Materials

With the piezoresistive technique, by imposing external force on a material, the resistance of the material dynamically changes according to the external force. Through constructing readout circuits and connecting them with material, the change in resistivity can be reflected through the changes in the output voltage [24]. Hence, the relationship between output voltage and applied force can be formulated, enabling the measurement of external force.

Chen et al. [25] and Gao et al. [4] have reported that piezoresistive representative materials consist of conductive ink, conductive polymers (fabrics and foams), and metal liquids. For these three kinds of materials, the process of achieving the piezoresistive effect varies. For conductive ink and polymers, the original materials are nonconductive; hence, their conductivity can be adjusted through methods such as doping fillers or heating. For metal liquids, which are already conductive, the major focus of the process is constructing metal gauge pieces with metal liquids because gauges produce different responses under external force in multiple directions.

In this section, we discuss these materials. As conductive fabrics and conductive foams are both conductive polymers, we discuss them together.

#### 2.1.1. Piezoresistive Sensing Mechanism of Conductive Polymers

To reflect a force with electric signals, piezoresistive sensing material should be conductive or semiconductive. Therefore, adjusting the conductivity of polymers is needed to transform polymers into piezoresistive sensing materials. According to the original conductivity, conductive polymers can be categorized into extrinsic and intrinsic polymers, each of which has different sensing mechanisms.

The processing of the extrinsic polymers focuses on doping the raw nonconductive material with a conductive material. When the concentration of added conductive fillers meets the percolation threshold, the conductive filler forms a percolation network that permits the electrons to move from one filler particle to another one [26]. This is also known as electron tunneling, which indicates the material has become conductive, which enables the conductivity of extrinsic polymers to be adjusted to achieve piezoresistivity [27].

**Figure 2:**
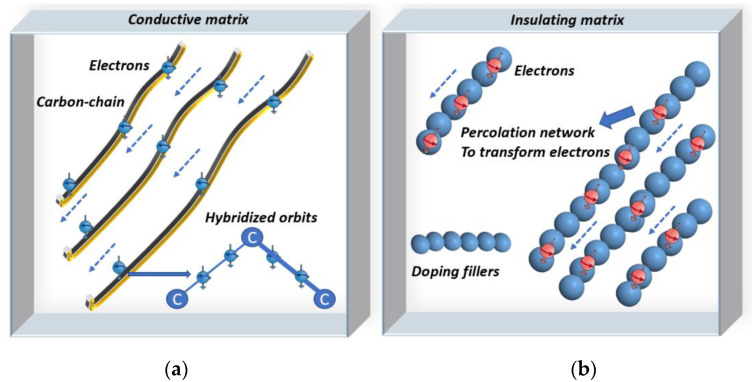
Conducting mechanism of intrinsic (**a**) and extrinsic (**b**) polymers. Electrons in hybridized orbits are released by bond-breaking methods such as heating and doping with fillers [27,28]. In extrinsic polymers, doping fillers form networks to transfer electrons [26].

Intrinsic polymers are named as such because of their hybridized orbits and special chemical bonds. Electrons connected by three sigma bonds can dwell in the p-z orbit. Thus, breaking the sigma bonds can release the restricted electrons, which can then transfer to the carbon chains. To achieve this purpose, suitable methods include doping particles into intrinsic polymers or heating. Therefore, the released electrons increase the conductivity of the material to achieve piezoresistivity [26,27,28]. Figure 2 illustrates the mechanisms of conductive intrinsic and extrinsic polymers.

#### 2.1.2. Piezoresistive Sensing Mechanism of Conductive Ink

The general types of conductive ink include carbon nanoparticles, metallic compounds, and metal nanoparticles [29]. In pressure sensors, carbon nanotubes and metallic compounds are most frequently applied [16,17]. On the one hand, the resistivity of metallic particles is relatively lower than that of conductive polymers [29], producing increased piezoresistivity. On the other hand, carbon nanotubes possess high flexibility and stretchability [29]. Therefore, compounds of carbon nanotubes and metallic particles are suitable for piezoresistive force sensors.

Processing conductive ink to attain the piezoresistive effect is simple. This can be achieved by heating the material because high temperatures break the metal–carbon composites into metallic particles, which can transfer electrons. Another method involves inserting special particles such as Au or Ag into the solvent at a particular ratio to produce a conductive liquid solution for further fabrication such as combining carbon nanotubes in [29,30]. Using these two methods, the raw material can become conductive or semiconductive.

#### 2.1.3. Piezoresistive Sensing Mechanism of Metal Liquids

Unlike conductive ink and conductive polymers, metal liquids are naturally conductive. They are usually fabricated into strain gauges for force sensing. The working mechanism of metal liquid is determined by strain gauges, which show different deformations under force in multiple directions. Strain gauges are embedded in a substrate material, instead of being tiled over the substrates. In most current products, S-shaped strain gauges are fixed in symmetry but in opposing angles [31].

**Figure 3:**
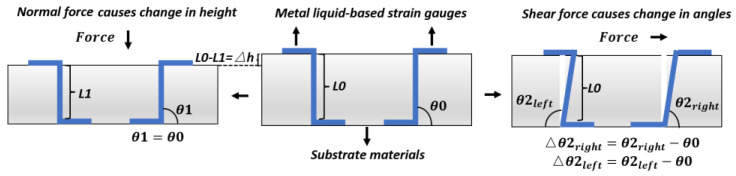
Response of metal liquid gauges under shear and normal forces [19,33]. Normal force causes vertical gauge strain, which is reflected in response height. Shear force causes gauge incline, which is reflected in response angle [31].

Constructing strain gauges with metal liquids enables the piezoresistive measurement of shear and normal forces [32]. Once an external force is applied, strain gauges present different deformations according to the direction of the force. For example, shear force causes strain gauges to show opposite deformation angles. With an applied normal force, they show the same deformation length, instead of being at angles to one another. Thus, their resistances alter differently [19,33]. As for the normal force, theoretically, when a normal force is applied, two gauges present the same change, and the output signals are the same [19,33]. The gauge-deforming process is demonstrated in Figure 3.

### 2.2. Piezoelectric Sensing Mechanisms and Suitable Materials

The phrase “piezoelectric effect” was first created in 1880 to describe the electric physical polarization phenomenon, which appears in the interaction between an external force and a non-centrosymmetric material [34]. The interaction process is principally influenced by the direction and strength of the external force. For instance, when vertically compressing a piezoelectric material, positive charges accumulate on the upper surface, while negative charges gather on the lower surface. Thus, polarization is formed. By connecting a polarized material with readout circuits, the electric signals reflect the change in applied force [35].

To quantify this effect, the following formula was established to describe the piezoelectric effect and correlation between polarization and imposed pressure:

(1) $$P_{i}=d_{ij}\sigma_{j}\;\mathrm{with}\;i=\;1,2,3\;\mathrm{and}\;j=\;1,2,3,4,5,6$$

where $P_{i}$ represents the polarization of the materials in direction i, $d_{ij}$ represents the piezoelectric strain factor, and $\sigma_{j}$ represents the strain in direction j. The degree of polarization reflects the magnitude of the force.

For most application scenarios, polarization and force both appear in 3-dimensional directions, so $d_{33}$ is most frequently used to assess the performance of a material and calculate the values of the force [36,37]. $d_{31}$ is also used for measuring shear stress.

The material has opposite responses to tensing and compressing [35]. This indicates that the piezoelectric effect is reversible, and this feature has enabled the design of energy harvest systems [36]. Hence, this kind of system is suitable for long-time gait feature supervision [38].

Since 1880, several kinds of material have been developed as the sensing material in piezoelectric sensors. They can be chiefly classified into four types: natural biological materials, natural crystals, piezoelectric polymers, and piezoelectric ceramics [39].

**Figure 4:**
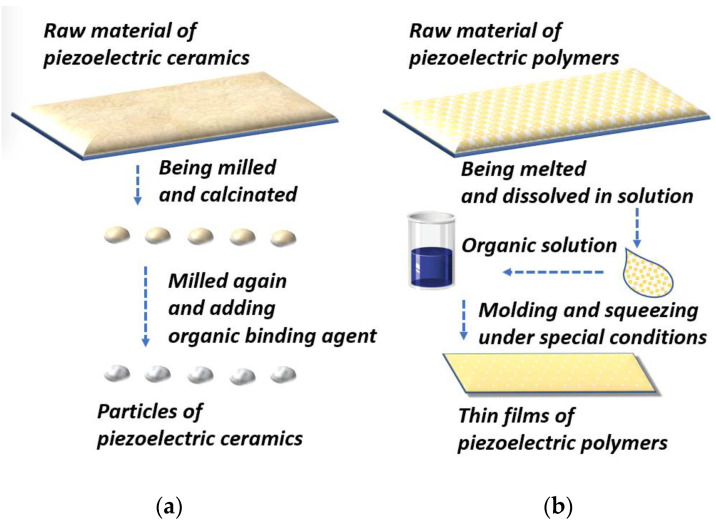
The fabricating mechanism of piezoelectric ceramics (**a**) and polymers (**b**). The key step of ceramics is milling into tiny size to achieve a more obvious piezoelectric effect [42,43]. While the key step of polymers is squeezing because its properties is decided in this stage [44,45].

To fabricate force sensors, piezoelectric polymers and ceramics are most frequently used because their properties can be adjusted in synthetic processes according to demands [39,40,41]. In applications, the PSD process often involves bending and deforming. This requires sensing material to be flexible and stretchable. Additionally, to attain a high $d_{33}$, materials should be small. To achieve these targets, piezoelectric ceramics are often milled into small particles, whereas piezoelectric polymers are usually transmuted into thin layers. We separately describe these processes in the following sections and Figure 4.

#### 2.2.1. Piezoelectric Ceramics

Piezoelectric ceramics are brittle and rigid and are formed by large particles. Thus, they cannot be directly applied in PSD sensors because the process of detecting plantar force often involves bending and deforming. Therefore, materials should be designed to have high stretchability and piezoelectric coefficients [25,42].

The ceramic most commonly used for piezoelectric force sensors is PZT (Pb[Zr(x)Ti(1−x)]O_3_). The manufacture of PZT can be divided into four steps: First, the raw PZT material is wet milled, which uniformly distributes the proportions of the ingredients. Second, the obtained particles are dried for further calcination, which is conducted in a pure chemical environment to prevent contamination. Third, after being calcinated under high temperature, for instance, around 1000 °C, the desired PZT phase is produced [43]. Fourth, the powders are milled again to ensure homogeneity and prepare them for the adjunction of an organic binding agent. Finally, the water in the composites evaporates, which indicates that the PZT for the piezoelectric sensor has been fabricated [43].

#### 2.2.2. Piezoelectric Polymers

The rigidity of ceramics hinders the stretchability of PSD sensors. To address this issue, organic piezoelectric polymer-based thin films were designed. Their merits include mechanical durability and flexibility. The most representative material is PVDF, which is the material we consider in the following paragraphs to demonstrate the physical manufacturing process of piezoelectric polymers.

The fabrication of PVDF can be briefly described as radically polymerizing monomer vinylidene difluoride at 10–150 °C and 10 to 300 atm. Polymerization can be divided into four steps [44,45]: First, the raw material is melted. Melted products are shaped in molds of the desired shapes at a relatively higher temperature. Second, the molded and cooled products are dissolved in a suitable solution to obtain the desired chemical properties. The solvent is evaporated from the composites, resulting in the final product having a porous shape. Third, the polymers are transformed into thin films. In this step, polymers are processed in a high-pressure environment and electric fields. Finally, the polymers are squeezed into thin films for further production of piezoelectric sensors [45,46,47].

## 3. Comparison and Review of Piezoresistive and Piezoelectric PSD Sensors

For pressure measurement, piezoresistive- and piezoelectric-based PSD insole systems have been fabricated for gait analysis. Their results in this task were reviewed in detail [25]. However, these two techniques have not yet been compared. In this study, we compared the systems in terms of material properties, sensing ability, and applications in gait monitoring. Our motivations for doing so were as follows:

First, we wanted to describe and compare the types of materials, and their characteristics, that can be exploited in PSD [48]. For example, the brittleness of PZT can provide a relatively higher $d_{33}$ for PSD. Second, the measuring ability of sensors is indicated by parameters such as sensing range, sensors size, and sensitivity. Their applicability in gait analysis is comprehensively determined by the sensor measuring ability and the features of the disease, so we compared these techniques from these aspects [5,7,25,49].

Based on the above, we reviewed and compared several piezoelectric and piezoresistive PSD sensors and present detailed tables and figures to demonstrate the comparison.

### 3.1. Review of Piezoresistive PSD Sensors for Gait Analysis

Generally, piezoresistive materials should have high mechanical flexibility and stretchability [48]. Thus, piezoresistive materials are widely used in elastic PSD sensors. Typically, the PSD sensor structure includes substrate materials, electrodes, and sensing material. Among these components, the sensing material is the most important, because its performance under an imposed force directly determines the sensor quality and applications. For example, carbon nanotubes and graphene-based sensors have high mechanical flexibility and stretchability; they can maintain high sensitivity under bending and pressing. Therefore, they can be used for PSD [4,5,7,12,22,23,24,25,26,27,28,29,30,31,32,33,34,35,36,37,38,39,40,41,42,43,44,45,46,47,48,49,50,51].

When an external force is applied, the change in force alters the resistance of sensing materials. The dynamic relationship between pressure and resistance can be observed through readout circuits [52,53]. During the moving period, sensors can capture the pressure-change process. Therefore, the value of and changing trend in plantar stress can be obtained. The results can assist in the diagnosis and analysis of chronic diseases such as Parkinson’s disease [54].

Currently, the several representative categories of the predominant piezoresistive materials are conductive organic polymers (fabrics and foam), conductive ink, and metal liquids [4,55,56,57]. We describe some of the current products of each kind of material in the following paragraphs. Most of the insole systems in examples were fabricated in the last 1–4 years, which represents recent materials and methods.

#### 3.1.1. Conductive Foam and Related PSD Sensors

For newly emerged piezoresistive PSD sensing layers, high flexibility and conductivity are the main requirements during the design process. Thus, using flexible polymers and salts in the design can simultaneously produce these two properties. For instance, TPU is a kind of elastomer that can be plasticized by heating and dissolved by solvents. By blending dissolved TPU with metal and salt compounds conductive foam mixtures can be produced. Thus, TPU-related materials can be used to produce piezoresistive sensors in insole systems.

Huang et al. [13] proposed an insole system equipped with a piezoresistive sensor matrix with 32 pressure sensors in a 4×8 matrix, each of which was 7.5×7.5 mm^2^. For their manufacture, they first dissolved the TPU with sodium chloride and CB in DMF; under the influence of the metal and salt, the obtained material was conductive. After the mixing, stirring, molding, and drying of the salt, the material obtained in the last stage was piezoresistive material for sensors.

By dissolving sodium chloride and volatilizing DMF, a sensor array with a multistage pore structure can be fabricated. This structure has satisfactory pressure sensitivity and other mechanical properties [58]. The experiment was divided into standing and walking phases to observe the response of the array in each situation. The results demonstrated that, in insole systems, the piezoresistive sensors can measure external pressure in the range of 20 Pa to 1.2 Mpa. This range is suitable for monitoring plantar pressure in normal life.

Although conductive foams and fabrics are both conductive polymers, conductive fabrics are more frequently used in force sensing because they can reduce the hysteresis effect to maintain sensitivity at a high level over a long working period.

A review [4] revealed that one disadvantage of piezoresistive sensors is hysteresis. Hysteresis has different definitions in the engineering domain. For insole systems, it refers to the issue where, after the removal of applied external pressure, the conductive property of the system does not return to the original state [30]. Therefore, when fitting the curve of external force and resistance, the trend, slope, and shape of the curve widely differ in the uploading and removing load stages. Hysteresis seriously decreases the sensitivity of sensors.

One way to overcome this issue is by repeating the experiment in dozens of cycles. By observing the curve each time, the formulation of hysteresis can be accurately calculated. Hence, the deviation of hysteresis is known when conducting further experiments.

Another method to address the hysteresis problem is choosing conductive fabrics as sensing materials for piezoresistive PSD sensors [14]. Due to the repeatability of cycles of experiments, this kind of material retains high sensitivity over a long working period. Therefore, these materials are a suitable option for piezoresistive sensors. For example, the insole system produced by Fei et al. [14] used RFP film as the conductive sensing material. They fixed eight round sensors at the metatarsals, phalanges, and heel. By testing the insole during running and walking, the results showed that the response curves of each sensor were stable as the number of steps increased. Thus, their sensors showed sustainable conductivity and did not experience hysteresis.

Adding metallic nanomaterials is also an option to enhance the stability of sensor sensitivity because they have high conductivity, like metal, and high flexibility and stability, like nanomaterials [59,60]. For instance, Zhang et al. used dicyclohexylcarbodiimide (DCCF) decorated with Ag nanowires (Ag-NWs) as the sensing material [61]. The sensitivity of the sensor was maintained at a high level (0.134 kPa) over 10,000 loading and unloading cycles. To increase sensitivity, Deng et al. designed a conductive fabric force sensor in a triple-layers structure [62]. They chose Ti3C2Tx MXene as the sensing material. In each sensing layer, the concentration of Mxene was inversely proportional to the resistance of the layer. In the triple-layer structure, they used the upper and lower layers as electrodes because of their low resistance. Hence, they coated the upper and lower layers with high-concentration Mxene and covered the middle layer with lower-concentration Mxene. Therefore, the middle layer became the sensing layer with high resistance. Their final product could measure pressure from 0.4 to 150 Kpa, with a sensitivity of 0.0034 Kpa. Although not yet used in PSD, this sensor’s performance indicates its huge potential for PSD.

These products proved their merits in force sensing; however, due to the potential toxicity of the materials, the conductive fabrics should be carefully selected.

#### 3.1.2. Conductive Ink and PSD Sensors

As the most common material used in piezoresistive PSD sensors, conductive-ink-related materials can be classified into carbon particles and metal nanoparticles.

As a carbon particle, carbon nanotubes have suitable strength and toughness and are extremely light. They also have both metal and semiconductor properties, which indicates that they have many potential applications in PSD. Composites of carbon nanotubes and carbon black are often used as the sensing material in PSD sensors [16,17]. An example of this is the insole system devised by Jung et al. [16]. In sensors in this system, the carbon black and multiwalled carbon nanotubes (MWCNTs) are functional nanopowders. To attain the best properties, they tried many ratios of carbon black to MWCNTs, finding an appropriate ratio of approximately 6:1. T PDMS was the substrate layer, which improved the plasticity of the material. During the fabrication, they used isopropyl alcohol (IPA) as a solvent, which finally evaporated to produce a porous structure. They eventually obtained four piezo-resistive sensors in the shape of a ribbon. Each sensor was 20 ×15 mm^2^. The results of the tension tests of the sensors showed that when the carbon black outnumbered the MWCNTs in a 6:1 ratio, the linear correlation coefficient between the change of resistance and resistance was approximately 0.95. They then applied the sensor for body weight estimation. The results of the experiments showed that the sensor could precisely measure a minimum weight change of 0.5–2.5 kg. As weight change is an aspect of some diseases such as diabetes, this product can be applied to daily body-weight monitoring for early diagnosis and prevention of diabetes. Furthermore, the monitoring data can help medical professionals to assess the effects of therapy [63,64].

For metal nanoparticles, adding metal particles into carbon nanotubes can promote the conductivity of sensing materials. Au is an option for this addition because it is conductive and stretchable and is chemically inert and so can increase the stability of sensors. As mentioned above, increases in repeatability and stability can reduce the negative effect of hysteresis. Additionally, altering the shape of a sensor containing Au is easier because of its high level of stretchability and malleability [65].

Zhao et al. [17] devised an Au/textile sensor system. Their sensor had two essential parts: substrate and sensing materials. They chose cotton fabric as the substrate and Au-NWs as the sensing material. They produced the sensor in two stages: forming Au-NW-impregnated fabric and fabricating piezoresistive sensors. The former stage involved blending Au-NWs and knitting the cotton into hexane to form an Au–fabric mixture. By repeating this procedure of knitting the mixture into hexane and evaporating the hexane dozens of times, the final Au-NW fabric mixture presented a stable black color. In the latter stage, they used electrodes with the Au-NW fabrics mixture to cover the substrates, thereby fabricating the prototype sensors. After conducting a lot of bending and stretching, the sensors showed flexibility and stretchability suitable for PSD. Then, they integrated the sensors under the toes and metatarsals of an insole. Their results showed that when bending the insole at different angles, the sensing range was 15 Kpa with sensitivity of approximately 0.29 Kpa.

**Figure 5:**
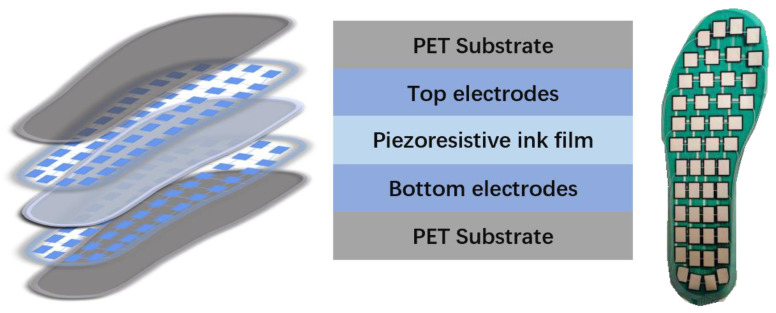
Structure and product of insole system proposed by Zhao et al. [11]. Sensing film is placed in the middle, while sensors are placed on the upper and lower film surfaces. Sensors are different sizes in different positions.

Due to the high flexibility and sensitivity of conductive ink films, PSD sensors fabricated with these inks can replace traditional PSD devices such as force plates. These sensors can be applied for diagnosing falling events, knee osteoarthritis (KOA), and similar diseases by detecting subtle pressure changes and deviations in the center of pressure (CoP) [66,67,68]. For instance, the conductive ink film-based PSD insole system proposed by Zhao et al. contains forty-eight sensors, as shown in the Figure 5. This system has successfully diagnosed KOA patients with an accuracy of 96.53% [11].

#### 3.1.3. Metal Liquids and Related PSD Sensors

For traditional piezoresistive PSD sensors, the detection of shear stress is also a problem. Hence, metal liquids were used because the strain gauges composed of metal liquids produce distinct responses under shear and normal forces. Although this type of material has not been widely used in PSD, their ability to detect shear force is suitable for PSD.

Shi et al. [19] proposed an EGaIn-based liquid metal as a sensing material for a piezoresistive sensor system. They chose polydimethylsiloxane (PDMS) as the substrate material. The manufacturing process of this material is complex: the key stage is screen-printing the liquid metal onto PDMS. Before the screen printing, two pieces of cured PDMS were prepared. The thicker piece was set to guarantee the adhesion of the liquid metal. The thinner piece was used to promote the coverage of the liquid metal. In the next stage, considering the relatively higher surface tension of liquid metals, they sprayed EGaIn liquid metal alloy as tiny droplets that fell on the substrate layer composed of PDMS. This stage was conducted under a high-pressure environment to guarantee that the liquid metal separated into droplets. The sensor was 2 mm tall and 4 mm wide. Each sensor had two resistors, each of which showed the same response under the impact of an external normal force. When shear force was applied, they showed different deformations according to the direction of the shear force. Thus, the sensors could simultaneously measure normal stress and shear stress.

For gait analysis, the ability to measure shear and normal stress was found to contribute to the diagnosis gait freezing [69], which is a representative clinical symptom of Parkinson’s disease. Additionally, changes in CoP were detected by detecting shear force [70]. Thus, such a sensor may also improve the prediction of falls. Moreover, through loading and releasing an external force at different speeds, the obtained curve between signal delay and speeds indicated that curves of loading and unloading overlapped. This finding indicated that liquid-metal-based piezoresistive sensors substantially reduce the hysteresis effect.

Wu et al. later altered the proportion of Ga and In to increase sensor sensitivity [71]. Furthermore, they used PDMS as the substrate material to accommodate EGaIn-based strain gauges, which prevented sensors from undesired moving deviation. In their sensor, Ga and In proportions were 68.5% and 21.5%, respectively; the remaining 10% of the sensing material was Sn. The sensitivity was 0.0168 Kpa.

The details of the products we described in Section 3.1 are presented in Table 1.

#### 3.1.4. Current Commercial Piezoresistive PSD Sensors

Piezoresistive PSD sensors are mostly designed and fabricated by laboratories and companies. Sensors produced by laboratories [6,72,73] are targeted for PD therapy and monitoring. These piezoresistive sensors can detect the occurrence of freezing of gait (FoG) by analyzing the distribution of plantar stress. Then, sensors send signals to ancillary devices, which deliver stimulation cues to patients to reduce the time of FoG. Thereby, the most important feature of these sensors is that they focus on a specific aspect of a disease.

Unlike laboratory-produced sensors, systems devised by companies concentrate on distinct goals. For instance, Tek-scan provides PSD sensors systems such F-scan^®^ and K-scan^®^. Orpyx devised the Orpyx-Si system. These products have some similarities; the main one is that most companies use multiwalled carbon nanotubes as the sensing material and PDMS as the substrate material. These systems apply sensors of various sizes, and the diameters of the sensors range from 3.81 to 80.9 mm. Insoles have four columns, with six to eight sensors in each column. In total, 24 to 48 sensors are used for PSD [11].

Because of their size and quantity, sensor arrays can cover most of the insole area, which also allows plantar stress to be detected in more places, increasing the sensing range. They can detect plantar pressure from 7 to 1043 Kpa [74,75,76,77]. This range is suitable for the diagnosis of some chronic diseases that involve higher peak plantar pressure. For instance, Organero et al. used eight force-sensing resistors (FSRs) to detect changes in peak plantar pressure. Then, they compared pressure between patients and normal people to diagnose KOA. The final accuracy of their system was 89% [78]. Another example is applications to stroke. In the system proposed by Howell et al., they used 32 FSRs to detect peak ground reaction pressure, which enabled the calculation of the swing and stance phases of the gait cycle [79]. As stroke patients usually have a shorter stance phase [80,81], this system can be applied to the monitoring of patient rehabilitation.

#### 3.1.5. Brief Summary of Piezoresistive PSD Sensors

Above, we introduced some recent studies on piezoresistive insole systems. We classified them into several types according to the type of sensing material. Each kind of sensor system has different advantages and drawbacks. In conclusion, the use of conductive-fabric-based sensors can reduce the hysteresis effect because of their higher repeatability, but their potential toxicity should be considered. Conductive-ink-based sensors have been most frequently used; these sensors are relatively more sensitive. Metal-liquid-based sensors are more sensitive, but this comes at the cost of higher hysteresis, and the fabrication procedure is more complex. For conductive foam, polymers are used to promote flexibility; salt is used to increase conductivity.

### 3.2. Review of Piezoelectric PSD Sensors for Gait Analysis

Generally, most piezoelectric PSD sensors are designed with a sandwich structure: electrodes–piezoelectric film–electrodes [82]. The main properties of sensors mostly depend on piezoelectric films.

The two main merits of piezoelectric PSD sensors are as follows: First, as introduced in Section 2, the reversible accumulation of charges on the upper and lower surfaces indicates that the energy can be harvested in dynamic working cycles. Second, the direction of the external force will determine the orientation of polarization. Therefore, the distinct responses of the material enable three-dimensional PSD. To describe three-dimensional PSD, the coefficients $d_{33}$ and $d_{31}$ are most frequently used in calculations.

Based on these merits, piezoelectric PSD sensors are suitable for applications that require a long working time and shear force detection. For instance, the prediction of falling events is mainly achieved through machine learning methods [83]. However, as the gait parameters are derived from activities of daily living, the obtained data are likely duplicate or near-duplicate samples [84]. Hence, this prediction requires numerous data from daily life to enable further analysis. For this purpose, sensors with low energy consumption are required.

Below, we provide examples of various piezoelectric PSD systems to illustrate the connection between piezoelectric techniques and gait analysis.

#### 3.2.1. Piezoelectric Ceramics and Related Piezoelectric PSD Sensors

As mentioned above, piezoelectric ceramics are milled into particles for further fabrication. The most common fabricating method is screen printing milled particles on a substrate layer under high temperature [85]. Sensors constructed with this method usually have a higher $d_{33}$. For instance, Son et al. [20] milled and screen printed three kinds of ECS-PolyPZT composite materials on alumina and polyimide substrates, which simultaneously produced conductive and flexible sensors. The results showed the value of $d_{33}$ was above 20 Pc/N, which could increase PSD sensitivity. However, these sensors still suffer drawbacks: they lack flexibility due to the large diameters (>2 μm) and rigidness of the particles [20,86]. Additionally, the complexity of the manufacturing process also hinders their wider application.

To address these issues, using smaller particles is a practicable solution. Altering the ingredients and proportions of materials is another option. The PZT-polymer insole system designed by Almusallam et al. [21] addressed the above-described problems. They used ferroperm as the sensing material. First, they milled the raw material into particles with diameters of 0.15, 0.3, and 0.8 μm. Second, they blended the particles with a polymer binder to ensure suitable flexibility. Third, they printed the mixture on the surface of polyurethane, which promoted bending flexibility. Finally, after heating the mixture until melting, they shaped the mixture into the desired shapes as plantar pressure sensors. They divided the insole into front and heel parts. Each part was covered by a sensor. The open-circuit voltage changed according to the dynamic influence of pressure generated by walking stages. Their results showed the changing trend in output voltage during the gait cycle. The detailed data showed that the sensitivity was 4 mV/N and $d_{33}$ was 36 Pc/N.

Given the sensitivity of these sensors, precise measurement of force distribution can be achieved. Therefore, this type of sensor can be used to monitor the plantar pressure distribution in the feet of diabetic patients to predict foot ulceration [87,88].

In addition to being placed over large areas of the insole such as the heel, PZT can be used as a sensing material for sensors under the toes. For example, Acer et al. proposed a sensor array composed of patterned electrode/PZT/patterned electrode in a sandwich structure. The sensor arrays were placed at the tips of each finger; the width and length of the sensor array were both 5 mm [89]. Through their experiments that involved monitoring dynamic changes in finger force, they found the sensitivity of their method was 0.821 V/N. For medical gait analysis, this sensitivity is suitable for detecting the diabetic ulceration of toes because [90] the forefoot and big toe are most likely to suffer ulcers, and the big toe generates higher plantar pressure than other toes. Therefore, the proposed sensors [89] can possibly be applied for diabetic foot ulceration diagnosis and prediction through monitoring.

In conclusion, to obtain higher sensitivity, piezoelectric-ceramics-based PSD sensors have smaller diameters and higher $d_{33}$ coefficients.

#### 3.2.2. Piezoelectric Polymers and Related Piezoelectric PSD Sensors

The above-described PZT-related studies have produced sensors with higher coefficients (>30 Pc/N) for pressure distribution measurement. However, the rigidness of ceramics complicates the manufacturing process because milling strong raw material into the desired shape is difficult. Additionally, although particles can be printed on electrodes, the lack of size precision poses an obstacle to production. Piezoelectric ceramics deform less than polymers due to their rigidity. This hence hinders the sensing range [20,21,89].

To address these issues, Rajala et al. [91] found that thin piezoelectric films must be used in future piezoelectric sensors: the use of thin films improves the flexibility of sensors during continuous walking and bending and their fabrication methods, such as lamination, are easier. As such, the representative polymer material, PVDF, had been applied [92]. Initially, PVDF-based force sensors were auxiliary in the capturing of movement. For instance, Xin et al. [93] used PVDF insole systems, with sensors composed of PVDF placed at the front of the sole and under toes. This flexible system could detect sudden changes during foot strike and toeing off the ground. Additionally, by combing IMU and PVDF PSD sensors, Zhou and Hu tracked the motion of arms [94]. The correlation between real motion and captured images was 98%.

**Figure 6:**
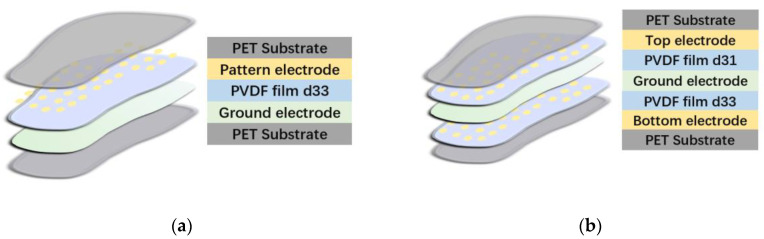
(**a**) Insole system [22], where one layer of PVDF was used; (**b**) insole system [12], where two different PVDF layers were used to detect shear and normal force.

Recently, PVDF has been used for PSD in different regions of the insoles. For instance, Dai et al. [22] constructed a simple insole system. As shown in Figure 6, in the middle of the sandwich structure, the most important layer was PVDF, which was 50 μm thick. In addition to PVDF, the electrodes were also essential for the quality of the system. To provide higher conductivity, the electrodes were composed of etching copper.

For fabrication, heating is conventionally the most frequent method of transforming PVDF into sensing layers. However, the high temperature decreases the sensitivity of the material. Therefore, the lamination technique was used in their manufacturing process, preventing the decrease in sensitivity in PVDF [95]. For the measuring stage, thirty-six sensors with a diameter of 4 mm were chosen to detect the accumulation of charge under plantar stress. The results showed that the sensitivity was approximately 0.69 mv/N and the detecting threshold was approximately 0.05 N, indicating the suitability of the sensor for monitoring subtle daily changes in plantar pressure. Three-dimensional force detection was not achieved because only one layer of PVDF was used, so only $d_{33}$ could contribute to PSD.

To increase accuracy and simultaneously measure shear and normal stress, an improved insole system was designed [12]. In the revised structure, as shown in Figure 6, two layers of PVDF were used: $d_{31}$ was the dominant coefficient in one layer and $d_{31}$ in the other. In each layer, the ratio of the dominant coefficient to the other was 10:1. The structure achieved 3D pressure detection because each layer responded differently to shear force.

The structures of these products are illustrated in Figure 6. The results showed that the sensitivity to normal plantar pressure and shear pressure was 56 and 174 mN, respectively, providing improvements on the standard (100 and 200 mN, respectively). Another advantage of these two insole systems is the promotion of IoHT: the data obtained from experiments can be observed in real time by researchers at laboratories or hospitals, and their decrease in sensitivity is less than 1.5% after 100 km of walking. They can provide precise data online.

Another representative type of piezoelectric polymer is polyaniline (PANI)-based composite material. PANI was originally used to mimic human skin. Materials for mimicking human skin are highly stretchable (140% to 180%) and have a large sensing range (1.8 MPa) [96]. Because of these characteristics, PANI-based material can be used in force sensing applications.

For instance, Wang et al. proposed a composite material composed of PANI, polyacrylic acid (PAA), and polyamide (PA). PA was used as the doping material to enhance the conductivity of the sensing layer, whereas PANI and PAA were used to mimic human skin. PANI makes the mimicked skin more robust because of its rigidness. PAA enables the skin to be more flexible because of its crosslinked elastin fibril [97]. Their product was highly stretchable (500%) and had a large sensing range (50 Pa to 1.5 MPa). Additionally, the fake skin takes advantage of the characteristics of piezoelectric materials, which is energy harvesting. Electrical properties (such as energy) can be restored within a day. Due to the high sensing range, stretchability, and energy-harvesting speed, this material can be used for PSD.

The products outlined in Section 3.2 are detailed in Table 2.

#### 3.2.3. Conclusions and Recent Commercial Progress of Piezoelectric PSD Sensors

Various studies have been conducted to improve the sensitivity, sensing range, and flexibility of sensor systems. Nevertheless, piezoelectric sensors have not yet been commercialized. The main reason for this is that polarization is aeolotropic. Thus, small external forces in each direction may cause noise in the sensor response. Thus, future studies should focus on removing the noise due to small forces whose direction is almost uncontrollable.

One solution to this issue is choosing a simpler fabricating method. This may be achieved by using a printing circuit board (PCB) to ensure the stability of parameters and simplify the fabrication procedures [12,22,99]. In one study, eight sensors were printed on piezoelectric films, which simplified the complex manufacturing process and stabilized the output current. The sensitivity and maximum detectable pressure were maintained at 69 Pc/N and 500 Kpa, respectively. The results showed that the sensitivity was maintained at a high level after 20,000 steps.

Lamination [98], PCB [12,22,99], and microfluidic spinning technology [100] can also be used to simplify fabrication. Meng et al. used this technique to construct β-phase-enriched PVDF microfibers [100]. This technique aims at inducing the inner PVDF solution [45] to enable phase transferring inside microfluidic channels. The obtained PVDF fibers had various diameters, which were woven into intertwined meshes to detect force from different directions.

Another solution is using microstructure materials. For example, Choi et al. [101] used micro-structured PDMS as the substrate material, which was bonded with a PZT piezoelectric layer to increase the stable sensitivity to 0.23 Kpa. Dayeh et al. used a micro-structured PDMS and ZnO thin-film transistors (TFTs) as piezoelectric channels. This design improved the ability to detect small forces as well because ZnO and TFT intrinsically enabled sensing and amplification of the forces [98].

Based on the new focus in recent studies, the theoretical foundation for commercial piezoelectric sensors has been laid.

### 3.3. Comparison of Piezoresistive and Piezoelectric Techniques

Above, we described the classification, suitable materials, mechanisms, and recent related insole systems. To summarize the information in this section, we briefly compare these two techniques in three aspects.

#### 3.3.1. Mechanisms

Both techniques derive from the same basic physical phenomenon. Pressure can be reflected through the changes in the resistance of piezoresistive materials under external force. Piezoelectric materials reflect pressure through polarization and reversal charge accumulation.

Their main difference is the methods through which the physical effects are produced. For piezoresistive materials, the conductivity of raw materials is adjusted as higher conductivity usually represents higher sensitivity, and most piezoresistive materials are naturally nonconductive [25,26]. For piezoelectric materials, polarization is an intrinsic feature of non-centered symmetric materials. The methods mainly concentrate on milling the material into films or particles to the desired size. These methods can also enhance the piezoelectric response [34,35,36,37,38].

#### 3.3.2. Main Advantages and Drawbacks

The distinct mechanisms have different advantages and drawbacks. For piezoresistive force sensors, changes in resistance are closely correlated with external forces in a certain direction [4,24]. Higher sensing range [16,17] and sensitivity [17,19] can be obtained by adjusting the conductivity. However, the modulus of sensing material increases with working time, which can decrease sensitivity and cause potential hysteresis [14,19]. Additionally, hysteresis and temperature [26,27] also decrease sensor accuracy.

For piezoelectric sensors, multidimensional and reversal polarization provides feasible energy harvest, which reduces power consumption and increases access for measuring shear force [12,22,98]. However, the dynamic working cycles can cause current leakage because static force prolongs the polarization time [102]. The complex structure [20,21] and noise caused by small forces are also concerns because these factors are closely related to shear stress detection.

#### 3.3.3. Chronic Diseases Suitable for Monitoring

According to the above advantages and drawbacks, we summarize the possible applications of these sensors. Piezoresistive PSD sensors have a higher sensitivity and wider sensing range, implying they are suitable for diseases with a higher peak pressure, longer stance phase, and whole-area detection, such as Parkinson’s disease, knee osteoarthritis, and stroke [103,104]. Some diseases require long-term monitoring to detect gradual issues, such as diabetic foot, so piezoelectric PSD sensors can be used due to their lower power consumption and longer working lifetime [105,106]. Furthermore, the center of plantar pressure (CoP) deviates in patients prone to falling and with flat foot. This CoP change is detected through detecting shear force, for which piezoelectric PSD sensors can be utilized.

**Figure 7:**
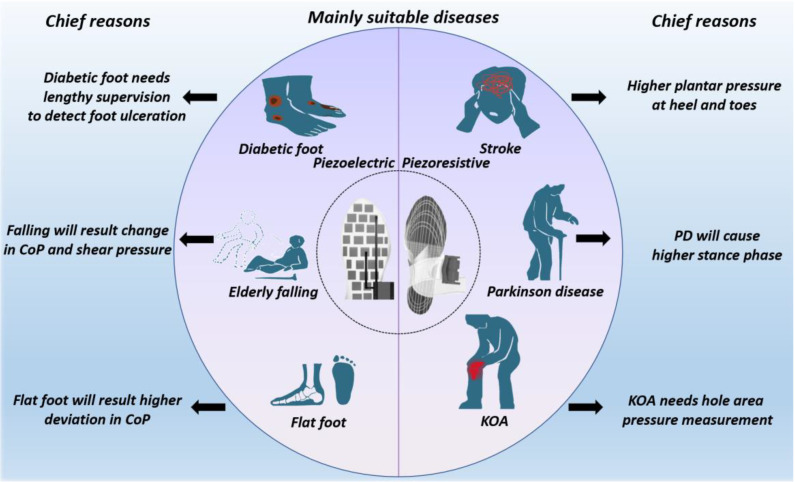
Diseases suitable for monitoring and why via two techniques: piezoresistive sensors are suitable for diseases requiring higher accuracy and larger detecting area, examples of which are stroke, PD, and KOA [103,104]. Piezoelectric sensors are suitable for diseases requiring longer working time and shear force detection, examples of which are diabetic foot, elderly falling, and flat foot [105,106].

The information is briefly summarized in Figure 7 and Table 3.

## 4. Challenges

As demonstrated by the studies described above, piezoelectric and piezoresistive pressure sensors have been applied for medical gait analysis. The features in the raw data were exploited to construct sensors, which have enabled convenient diagnosis and increased accuracy of measurement. Nevertheless, some challenges still hinder the wider use of piezoelectric and piezoresistive sensors. A brief summary of the challenges is demonstrated in Table 4.

### 4.1. Application Challenges

For medical purposes, ideal insole systems should be sustainable, which means the sensing accuracy should be maintained at a high level over a long working period. However, current sensors have limited working sustainability.

For piezoresistive materials, their decrease in sensitivity is the main problem. When the external force increases, the modulus of the insole material increases as well. Hence, the deformation of materials under the same force declines according to Hook’s law. The decreased sensitivity influences diagnosis accuracy. For instance, due to the damage to their plantar nerves, patients with diabetes present clinical features such as higher peak plantar pressure. However, the result obtained from the sensors includes pressure imposed by users and the deviation of the sensor. Hence, the early prediction accuracy is lower.

For piezoelectric materials, plantar stress is detected based on dynamic gait cycles. For some clinical manifestations, such as freezing of gait (FoG), the foot of patients stagnates for a short time interval. However, under the influence of static force, current leakage leads to decreased pressure detection accuracy. Therefore, the working efficiency of piezoelectric materials is lower in some static or quasistatic scenarios such as FoG.

### 4.2. Limitations of Sensing Materials

Although the sensitivity, sensing range, and working lifetime of sensors have been improved in recent studies, the intrinsic features of sensing materials prevent further application.

The challenge common to these two techniques is that sensing materials are both sensitive to temperature. For piezoresistive materials, increases in temperature increase the resistance of metal material while considerably decreasing the resistance of semiconductors [26,27,28], because the heating or cooling process will sharply increase the conductivity of sensing materials. Piezoelectric materials are sensitive to temperature changes due to the pyroelectric effect. For asymmetric crystals, the number of polar charges on the surface gradually decreases with increasing temperature [95].

The materials in these two techniques also have their own limitations.

Piezoresistive materials are mostly elastic materials, so hysteresis is inevitable. When the external force is removed, if the deformation recovery time is longer than the average time per step, the measurement accuracy and sensitivity of PSD decreases [4,30]. Additionally, the inability to detect shear force is a shortcoming, as mentioned in [31,32]. Though metal liquid-based sensing material can overcome this problem, it has not been widely used.

For piezoelectric materials, there is more than one piezoelectric coefficient in a film of piezoelectric material, which means that both $d_{33}$ and $d_{31}$ exist [12,22]. Therefore, a piezoelectric material with a dominant coefficient in measuring force in a single direction is not as accurate as a piezoresistive material, because another piezoelectric co-efficient can interfere with the measurement results. Though this effect can be used to measure shear force and normal force simultaneously [12], the co-existing phenomenon will influence the accuracy of measurement.

## 5. Future Research Directions

In the foreseeable future, we think that these two techniques can be combined with the following technologies to advance PSD and associated healthcare applications.

### 5.1. Multi-Sensing-Based Self-Calibration Function

To overcome the degradation in performance caused by environmental factors, the traditional solution is manual calibration. However, frequent manual calibration is not beneficial for wider applications. As each sensing material is subject to different environmental influences, self-calibration can be achieved by simultaneously using multiple sensing techniques. The combined use of multiple technologies can help improve the performance of sensor systems.

Piezoresistive materials are less affected by temperature than piezoelectric materials, so the change in piezoelectric coefficient can be calculated by comparing the detection results in the vertical direction. This self-calibration can specifically be realized because the output voltage of the $d_{31}$ layer is jointly generated by shear and normal stress. Therefore, by subtracting the output voltage of the piezoresistive sensor from the output voltage of the $d_{31}$ layer, the voltage corresponding to the accurate shear stress component can be obtained.

### 5.2. Digital Twin (DT) Technology

DT technology involves the duplication of objects, which can be observed in virtual environments. In the medical domain, the construction of DTs for the human body is one of the future trends in healthcare. Through constructing human DTs, medical professionals can not only diagnose current diseases but also predict potential disease development trends [107]. Furthermore, based on DT data, professionals can formulate suitable rehabilitation strategies for a patient [108].

In the establishment of human DTs, wearable devices are most frequently used to obtain data. Among all kinds of wearable devices, gait-based sensors are more suitable because gait is generated from the coordination between nerves, muscles, and lower limbs. Hence, these data can more comprehensively reflect lesions. Additionally, gait detection does not affect the normal life of users. Therefore, gait detection is one of the most convenient methods to obtain large amounts of comprehensive data [25,108,109,110].

Although piezoresistive and piezoelectric techniques are the most commonly used to detect plantar pressure, plantar force is not the only gait parameter most closely related to lesions. Therefore, piezoresistive and piezoelectric PSD sensors can be combined with sensors, such as inertial measurement unit (IMU) and electromyogram (EMG) sensors, in other locations to obtain data for establishing DT models, thereby providing a more accurate basis for disease detection and rehabilitation monitoring.

## 6. Conclusions

In this study, we first analyzed the current situation of mainstream piezoresistive- and piezoelectric-based PSD sensors in gait analysis. Second, we introduced the recently developed advanced piezoresistive and piezoelectric materials and explained their potential use in improving PSD performance.

In Section 2, we explained the processes of fabricating representative piezoelectric ceramics and polymers and described the conducting mechanisms of recent piezoresistive materials, including conductive ink, polymers, and metal liquids. We provided readers with a review of their working principles. In Section 3, we reviewed various studies and results based on recently used materials.

In Section 4, we analyzed the current drawback, which is the limited working sustainability, of these two kinds of PSD sensors due to their sensing mechanisms.

Finally, we proposed future trends in these techniques. These two techniques can be used together to achieve self-calibration, which can enhance sensitivity and working efficiency. These techniques should also be combined with other wearable devices to establish DT models of users, which can provide medical professionals with more detailed information for diseases diagnosis. 

## Figures and Tables

**Table 1:** Brief description of sensors outlined in Section 3.1. Conductive ink-based sensors are used more often because of their higher sensitivity and stretchability. Other types of materials have diverse merits such a non-hysteresis, simple fabrication, and ability to detect shear force.

Type of Material	Sensing Material	Parameters	Main Advantages and Drawbacks
Conductive foam [13]	TPU	Range: 20 Pa–1.2 Mpa	Simpler fabrication
	CB	32 sensors in a matrix	Higher stretchability
	NaCl	Sensor size: 7.5×7.5 mm^2^	Higher conductivity
Conductive fabrics [14]	RFP film	Range: 0–400 N	Higher repeatability
		8 round sensors	Lower hysteresis
		Sensor diameter: 8 mm	Potential toxicity
Conductive ink [16]	PDMS	Sensitivity: 0.5–2.5 Kg	Wider use
	MWCNT	4 sensors in a matrix	Higher sensitivity
		Sensor size: 20×15 mm^2^	Higher plasticity
Conductive ink [17]	PDMS	Sensitivity: 0.29 Kpa	Higher sensitivity
	Au-NWS	Range: 0–15 Kpa	Higher stretchability
Metal liquids [19]	EGaIn PDMS	Sensitivity: 2N Sensor size: 2×2 mm^2^	Higher hysteresis Higher sensitivity Shear force detection

**Table 2:** Brief description of sensors in Section 3.2. Piezoelectric ceramics have larger particle diameters and sizes, but their stretchability and sensitivity need to be improved. Piezoelectric polymers have higher stretchability and stronger ability to detect shear stress.

Type of Material	Utilized Material	Parameter	Main Advantages and Drawbacks
Ceramics [20]		Diameters of particles: ≥ 0.3 μm	High conductivity
	ECS-PolyPZT Al	$d_{33}$ = 29 Pc/N	Medium flexibility Larger particle diameters
Ceramics [21]	PZT Ferroperm	Diameters of particles < 0.3 μm $d_{33}$ = 36 Pcma/N Sensitivity: 0.4 Mv/N	High flexibility More complex production Smaller particle diameters
Polymers [22]	Copper	Sensitivity: 0.056 N	Only normal stress
	PVDF Patterned electrodes	$d_{33}$ around 60 Pc/N	Higher sensitivity Simpler fabrication
Polymers [12]	Copper	Normal sensitivity: 0.056 N	Higher and wider sensitivity
	2 layers of PVDF Patterned electrodes	Shear sensitivity: 0.174 N $d_{33}$ and $d_{31}$ around 60 Pc/N	Higher stability Simpler use
Micro-structure Materials [98]	ZnO/PZT PDMS as substrate layer	Sensitivity: 0.293 Kpa $d_{33}$ = 69 Pc/N Sensing range: 0.2–500 Kpa	Higher coefficients Simpler fabrication Higher sensing range

**Table 3:** Comparison of two techniques’ mechanisms: advantages and drawbacks. Each technique has two separate and typical mechanisms. Advantages and drawbacks were derived from mechanisms.

Technique	Basic Mechanisms	Main Advantages	Main Drawbacks
	Changing resistivity under external force. [25,26]	Near linear correlation;	Changing modulus decreases accuracy in walking. [14]
Piezoresistive		static force measurement. [16,17]	
Sensing	Adjusting conductivity to form piezoresistive material. [4,24]	Higher sensing range; higher sensitivity. [16,17,19]	Potential hysteresis; [14,19] sensitive to temperature. [26,27]
	Polarization under external force in each direction. [34,37]	Shear force measurement;	Complex fabrication
Piezoelectric		simpler structure. [22,98]	sensitive to small forces. [20,21]
Sensing	Reversal charges accumulation under impact of force. [35,36]	Feasible charges harvest;	Potential current leakage under static force. [102]
		less energy consumed. [12]	

**Table 4:** A comparison of challenges and correspondent reasons of two techniques.

Technique	Challenges	Reasons
Piezoresistive	Decreasing sensitivity in utilization [4,30]	Hysteresis will decrease accuracy during utilization [30]
Sensing	Sensitive to temperature change [26,27,28];	Temperature changes conductivity [26,28]
	limited detecting directions [31,32]	Materials cannot detect shear force [31,32]
Piezoelectric	Influencing accuracy in static PSD [12,22]	$d_{33}$$\;\mathrm{and}\;d_{31}$ both exist in one PVDF layer [12,22]
Sensing	Sensitive to temperature change [95]	The pyroelectric effect will decrease the charges on the surface [95]

## Data Availability

Not applicable.
